# Interpretable machine learning-derived nomogram model for early detection of diabetic retinopathy in type 2 diabetes mellitus: a widely targeted metabolomics study

**DOI:** 10.1038/s41387-022-00216-0

**Published:** 2022-08-05

**Authors:** Jushuang Li, Chengnan Guo, Tao Wang, Yixi Xu, Fang Peng, Shuzhen Zhao, Huihui Li, Dongzhen Jin, Zhezheng Xia, Mingzhu Che, Jingjing Zuo, Chao Zheng, Honglin Hu, Guangyun Mao

**Affiliations:** 1 Division of Epidemiology and Health Statistics, Department of Preventive Medicine, School of Public Health & Management, Wenzhou Medical University, Wenzhou, Zhejiang China; 2 Center on Evidence-Based Medicine & Clinical Epidemiological Research, School of Public Health & Management, Wenzhou Medical University, Wenzhou, Zhejiang China; 3 Department of Medical Statistics and Epidemiology, School of Public Health, Sun Yat-sen University, Guangzhou, Guangdong China; 4 Center on Clinical Research, School of Ophthalmology & Optometry, Wenzhou Medical University, Wenzhou, Zhejiang China; 5 The Second Affiliated Hospital of Zhejiang University School of Medicine, Hangzhou, Zhejiang China; 6 Department of Endocrinology, the First Affiliated Hospital of Anhui Medical University, Hefei, Anhui China

**Keywords:** Risk factors, Diabetes complications

## Abstract

**Objective:**

Early identification of diabetic retinopathy (DR) is key to prioritizing therapy and preventing permanent blindness. This study aims to propose a machine learning model for DR early diagnosis using metabolomics and clinical indicators.

**Methods:**

From 2017 to 2018, 950 participants were enrolled from two affiliated hospitals of Wenzhou Medical University and Anhui Medical University. A total of 69 matched blocks including healthy volunteers, type 2 diabetes, and DR patients were obtained from a propensity score matching-based metabolomics study. UPLC-ESI-MS/MS system was utilized for serum metabolic fingerprint data. CART decision trees (DT) were used to identify the potential biomarkers. Finally, the nomogram model was developed using the multivariable conditional logistic regression models. The calibration curve, Hosmer–Lemeshow test, receiver operating characteristic curve, and decision curve analysis were applied to evaluate the performance of this predictive model.

**Results:**

The mean age of enrolled subjects was 56.7 years with a standard deviation of 9.2, and 61.4% were males. Based on the DT model, 2-pyrrolidone completely separated healthy controls from diabetic patients, and thiamine triphosphate (ThTP) might be a principal metabolite for DR detection. The developed nomogram model (including diabetes duration, systolic blood pressure and ThTP) shows an excellent quality of classification, with AUCs (95% CI) of 0.99 (0.97–1.00) and 0.99 (0.95–1.00) in training and testing sets, respectively. Furthermore, the predictive model also has a reasonable degree of calibration.

**Conclusions:**

The nomogram presents an accurate and favorable prediction for DR detection. Further research with larger study populations is needed to confirm our findings.

## Introduction

Diabetic retinopathy (DR) is the most common microvascular complication of diabetes mellitus (DM) and a leading cause of visual impairment in working-age adults worldwide [1, 2]. It was estimated that 191 million people would be diagnosed with DR by 2030 [3]. The economic impact of DR is huge, tripling the medical costs of DR patients compared with ordinary diabetics, and visual impairment and blindness can also have a devastating impact on personal quality of life and the socio-economic conditions in which they live [4–7]. In the United States alone, DR-related blindness may generally cost up to $500 million per year [8].

DR can be classified into two stages, non-proliferative DR (NPDR) and proliferative DR (PDR). In clinical practice, the standard treatments, including laser photocoagulation, intravitreal injections of corticosteroids or anti-VEGF agents, and vitreoretinal surgery, mainly target the late stage of DR [9]. Treatments mentioned above are quite expensive, all require a vitreoretinal specialist and induce several side effects [9]. In this condition, the vision of many DR patients would be severely affected, in which some irreversible damages have occurred [9]. Therefore, timely recognition of DR at its early stage is essential for those who need comprehensive ophthalmological examination and treatment to avoid permanent vision loss [10].

For most complex diseases, there exists a long-term duration from the exposure to clinical manifestations, which generally includes 3 stages: normal state, pre-disease state, and disease state [11]. It is reported that from the pre-disease to early-disease states, levels of many internal bio-signals, including endogenous small metabolites, vary dramatically and remain stable to the clinical stage. These apparent alterations can be instinctively observed by some sensitive assessments. Routine screening methods for DR that include fundus photography can detect retinal hemorrhage, exudates, neovascularization, and angioma formation. Nowadays, it has become an accurate, cost-effective, and reproducible method for DR diagnosis. However, this is only possible when DR has progressed into the clinical stage and has some clinical evident symptoms and signs, which is too late for DR’s effective prevention and control.

In the past several decades, a variety of new technologies have been proposed constantly, which have highly changed traditional screening strategies. Among them, metabonomic studies and machine learning (ML) algorithms are commonly utilized for finding potential biomarkers and automatic detection or classification of DR. In fact, metabonomics can comprehensively capture and systematically analyze the spectrum of various metabolites in the process of disease development and has great advantages in elucidating abnormal sites, disease occurrence, the development mechanism, and early recognition. However, the metabonomic data are abundant and complex with high dimensions, which bring challenges to the traditional data preprocessing methods and statistical analysis [12]. It is widely accepted that ML algorithms can effectively use various, huge or complicated data to train models and make decisions for certain performance indicators. Due to the ability to find complex patterns in high-dimensional and heterogeneous data, ML has become an important tool for understanding genomic as well as metabolome data [13].

Based on the propensity score matching approach (PSM), the current study aimed to screen signature biomarkers associated with DR occurrence utilizing metabolomics techniques as well as machine learning algorithms and to develop an ideal nomogram model for predicting the personalized likelihood of DR in type 2 diabetic patients.

## Methods

### Subjects

This is a propensity score matching (PSM) based case-control study. Of the 950 participants enrolled from the second affiliated hospital of Wenzhou Medical University (WMU) and the first affiliated hospital of Anhui Medical University (AMU), 755 were healthy volunteers, 112 were determined as type 2 diabetes mellitus (T2DM) without DR, and 83 were diagnosed as DR during August 2017 to June 2018 (Fig. S1). All recruited participants had received careful ophthalmic examination and taken retinal photographs by two independent trained ophthalmologists. Fundus color photographs were taken using a Non-Mydriatic Retinal Camera from TOPCON TL-230D, Japan. If the two ophthalmologists had different diagnostic opinions, they were handed over to the third chief physician for the final diagnosis.

The inclusion criteria were as follows: (1) T2DM; (2) ≥35 years old; (3) voluntary participation and signed informed consent form. Participants with the following situations would be excluded: (1) any other eye diseases or history of eye surgery; (2) cancer, infectious disease, mental disorder, heart failure, severe hypertension (systolic blood pressure ≥180 mm Hg or diastolic blood pressure ≥110 mm Hg) and any other severe chronic systemic disease; (3) poor quality of fundus photographs, which were not clear for DR diagnosis. Only those following each of the inclusion criteria and none of the exclusion criteria were potential participants.

To avoid various types of bias due to confounding factors or uneven data distribution, PSM was used to match the DR and DM groups according to a 1:1 ratio for age, sex, body mass index (BMI) and glycated hemoglobin (HbA1c), and to match the DM and healthy control groups were matched for age, sex and BMI, and 69 blocks were obtained for inclusion in the analysis. The sample size was sufficient to meet statistical requirements and guarantee the reliability of our findings, and the sample size and power estimations were specifically described in our previous studies [14, 15].

The 69 blocks were randomly divided into a training set and a test set according to the principle of independent homogeneous distribution in the ratio of 7:3, and the former was used for the construction of the early identification model, and the latter was used for the evaluation of the model identification effect. Before the beginning of this study, the protocol had been approved by the Ethics Committee of the Eye Hospital of WMU [Number: KYK (2017) 46] and confirmed by the two associated hospitals. All participants in the current study were voluntary and had provided written informed consent.

### Serum collection and preparation

A detailed description of this part could be found in our previous work [16]. In brief, participants were fasted for at least 8 (8–10) hours before obtaining blood samples, which were collected between 8:00 and 10:00 in the morning from study participants. The samples were thawed on ice and three volumes of ice-cold methanol were added to one volume of serum. The mixture was whirled for 3 min and centrifuged with 12,000 r/min at 4 °C for 10 min. The supernatant was collected and centrifuged at 12,000 r/min at 4 °C for 5 min. Finally, the supernatant was collected again for the following determination of metabolites via ultra-high performance liquid chromatography-electrospray ionization tandem mass spectrometry (UPLC-ESI-MS/MS) system.

### UPLC-ESI-MS/MS metabolomics profiling

The endogenous small molecule metabolites in the collected supernatant were carefully assessed by trained professional technicians in the central laboratory using the UPLC-ESI-MS/MS system (UPLC, Shim-pack UFLC SHIMADZU CBM A system, https://www.shimadzu.com/; MS, QTRAP® 6500+ System, https://sciex.com/). The analytical conditions were as follows: UPLC: column, Waters ACQUITY UPLC HSS T3 C18 (1.8 µm, 2.1 mm × 100 mm); column temperature, 40 °C; flow rate, 0.4 mL/min; injection volume, 2 μL; solvent system, water (0.04% acetic acid): acetonitrile (0.04% acetic acid); gradient program, 95:5 V/V at 0 min, 5:95 V/V at 11 min, 5:95 V/V at 12 min, 95:5 V/V at 12.1 min, 95:5 V/V at 14 min. Linear ion trap (LIT) and triple quadrupole (QQQ) scans were acquired on a triple quadrupole-linear ion trap mass spectrometer (QTRAP), QTRAP® 6500+ LC-MS/MS System, equipped with electrospray ionization (ESI) Turbo Ion-Spray interface, operating in positive and negative ion mode and controlled by Analyst 1.6.3 software (Sciex). The ESI source operation parameters were as follows: source temperature 500 °C; ion-spray voltage (IS) 5500 V (positive), −4500 V (negative); ion source gas I (GSI), gas II (GSII), curtain gas (CUR) were set at 55, 60, and 25.0 psi, respectively; the collision gas (CAD) was high. Instrument tuning and mass calibration were performed with 10 and 100 μmol/L polypropylene glycol solutions in QQQ and LIT modes. A specific set of multiple reaction monitoring transitions was monitored for each period according to the metabolites eluted within this period.

### Data preprocessing

The characteristic departure of each substance was screened by the triple quadrupole, and the signal strength of the characteristic departure was obtained. For the extraction of all metabolites, the off-peak peaks were integrated into the sub-peak integral respectively, and the “spectral peak advance” integral of the same metabolites in different samples was corrected [17].

**Fig. 1:**
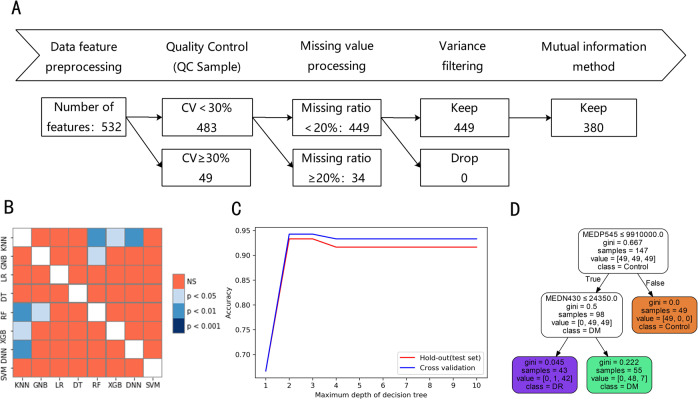
Data preprocessing and selection of machine learning models. Metabolomic data preprocessing work flow (**A**), accuracy heat map of machine learning model (**B**), decision tree (**D**), and its hyper-parameter learning curve (**C**). Notes: **C** Maximum depth parameter (max_depth) selection in the decision tree model used hold-out and 10-fold cross-validation based on the hyper-parameter learning curve; **D** A decision tree model based on the training set to distinguish the healthy control group, DM group, and DR group. Abbreviations: QC quality control, CV Coefficient of variation, KNN K-Nearest Neighbors, GNB Gaussian Naive Bayes, LR Logistics Regression, DT Decision Tree, RF Random Forest, XGB XGBoost, DNN Neural Networks, SVM Support Vector Machine, MEDP545 2-pyrrolidinone, MEDN430 thiamine triphosphate, Control healthy control group, DR diabetic retinopathy group, DM diabetes mellitus without DR group.

To assess the reliability of the determination, quality control (QC) samples were prepared by mixing the samples to be tested in advance. One QC sample was inserted into the determination sequence at every 20 samples to be tested. Metabolites associated with the coefficient of variation (CV) >30% in the QC samples were excluded in the following data analysis [18]. Features with missing values of <20% were filled with half of the lowest detected values [18]. Otherwise, they would be discarded [19]. Furthermore, the approach of variance filtering was performed and features with variances equal to 0 were also deleted. In addition, we used a mutual information method to capture correlated features (either linear or nonlinear relationships) (Fig. 1A).

### Statistical analysis

#### Clinical examination data

Continuous variables with normal distribution or approximately normally distributed were described as mean ± standard deviation (SD), and analysis of variance for a randomized block design or the paired *t* test was applied to compare the differences among the cases and controls. Continuous variables not normally distributed were described as median (1st quartile, 3rd quartile), and Friedman M or Wilcoxon signed-rank tests were used to comparing across groups. Categorical data were presented as the number of cases (%) and the McNemar-Bowker test was utilized to assess the comparability of the groups.

#### Metabolomics-based indicator detection

To identify the prognostic biomarkers and develop a predictive model, we performed eight different ML algorithms including K-Nearest Neighbors (KNN), Gaussian Naive Bayes (GNB), Logistics Regression (LR), Decision Tree (DT), Random Forest (RF), XGboost (XGB), Neural Networks (NNs) and Support Vector Machine (SVM). The 10-fold cross-validation was used as a model evaluation strategy to avoid over-fit. The parameters of each model were optimized by the related learning curve and grid search approach. A Nonparametric Friedman test was used to compare the performance of the eight machine learning models mentioned above. When the test was statistically significant (*p* < 0.05), an additional Nemenyi test was used to conduct further post hoc analysis. The best model was screened by the comprehensive comparisons of each model’s performance, simplicity, and interpretability. In the end, metabolic biomarkers were determined based on the final predictive model.

#### Interpretable machine learning-derived nomogram model construction

To effectively distinguish DR cases from T2DM patients, a combined predictive model including detected differential metabolites and some clinical indicators was established based on multiple conditional logistic regression and presented with a nomogram. Moreover, we used the area under the curve (AUC) of the receiver operating characteristic (ROC) analysis to evaluate its discrimination of the nomogram, and the calibration curve and Hosmer–Lemeshow test to evaluate the calibration. In addition, the nomogram’s clinical utilities were also carefully investigated using decision curve analysis to make up for the ROC curves’ limitations, which could not achieve the best sensitivity and specificity at the same time. Furthermore, potential overfitting was also well considered in the current study. Finally, the likelihood of DR for each participant was estimated by the nomogram based on the training set, and extensively validated in a separate testing set, respectively.

According to the principles of data mining and predictive model construction, the dataset was randomly split into a training set and another independent testing set, at a ratio of 7:3, using the train_test_split approach in scikit-learn package of Python 3.8.7 (Copyright © 2001–2020 Python Software Foundation). All data management and statistical analyses were carried out using Stata/MP 15.1 for windows (Copyright 1985–2017 StataCorp LLC, College Station, Texas 77845 USA). Figures were drawn with Python 3.8.7 and R-Studio for windows (Version 1.4.1103 © 2009–2021 Rstudio, PBC). All tests were two-sided and *p* value < 0.05 was set as the significant level.

## Results

### Study participants determination and their characteristics

Depending on the PSM approach, a total of 69 blocks, comparable in clinical characteristics except for systolic blood pressure (SBP) and duration of diabetes, were obtained. The mean age of the matched participants was 56.7 years with a standard deviation of 9.2, and 61.4% were males. Among those 69 DR patients, 9 (13.0%), 31 (44.9%), 20 (29.0%), and 9 (13.0%) were classified into mild, moderate, severe NPDR and PDR, respectively. The demographic and clinical characteristics of the participants are given in Table 1.

**Table 1:** Clinical and demographic characteristics of the study population.

	Training set			Testing set		
Variables	Control	DM	DR	Control	DM	DR
Age, years	56.0 (54.0,62.0)	53.0 (48.0,59.0)	57.0 (54.0,65.0)	58.3 ± 7.0	56.0 ± 12.0	57.0 ± 10.0
Male, # (%)	36 (73.5)	29 (59.2)	26 (53.1)	17 (85.0)	9 (45.0)	10 (50.0)
BMI, kg/m^2^	24.5 (23.4,26.7)	23.9 (22.1,27.4)	24.1 (22.4,27.0)	24.7 (23.2,26.9)	24.8 (23.1,26.1)	25.2 (22.4,26.2)
Fpg, mmol/L	5.4 (5.0,5.7)	8.3 (6.9,12.0)	8.9 (6.7,10.9)	5.3 (5.1,6.4)	8.6 (6.8,11.6)	7.3 (5.7,9.1)
HbA1c, %	5.7 (5.4,6.0)	9.9 (8.2,12.0)	9.7 (8.7,10.9)	5.9 ± 0.8	9.9 ± 2.0	10.3 ± 1.9
LDL, mmol/L	2.9 ± 0.8	2.7 ± 0.9	2.6 ± 1.1	2.7 ± 0.7	2.4 ± 1.2	2.5 ± 1.0
HDL, mmol/L	1.1 (1.0,1.3)	1.1 (0.8,1.4)	1.1 (0.9,1.4)	1.1 (1.0,1.2)	0.9 (0.8,1.2)	1.0 (0.8,1.2)
TG, mmol/L	2.0 (1.5,3.0)	1.6 (1.0,2.1)	1.4 (1.0,1.7)	1.9 (1.4,2.7)	1.8 (1.5,2.4)	1.5 (1.1,2.3)
TC, mmol/L	5.0 ± 0.9	4.8 ± 1.1	4.6 ± 1.5	4.9 ± 0.9	4.5 ± 1.3	4.3 ± 1.3
hypertension, # (%)	17 (34.7)	12 (24.5)	21 (42.9)	2 (10.0)	10 (50.0)	10 (50.0)
Systolic BP, mm Hg		124.8 ± 14.4	136.8 ± 19.7		134.5 ± 17.6	140.1 ± 24.8
Diastolic BP, mm Hg		78.0 (73.0,86.0)	76.0 (70.0,80.0)		82.6 ± 10.3	80.6 ± 11.5
Duration of diabetes, years		8.1 ± 6.2	12.2 ± 6.1		9.9 ± 6.6	12.1 ± 7.8
Education, # (%)						
Junior high school or below		25 (53.2)	23 (51.1)		10 (52.6)	13 (65.0)
High school or above		22 (46.8)	22 (48.9)		9 (47.4)	7 (35.0)
Occupation, # (%)						
Manual workers		23 (48.9)	22 (50.0)		8 (44.4)	12 (60.0)
Mental worker		10 (21.3)	8 (18.2)		5 (27.8)	3 (15.0)
Both		14 (29.8)	14 (31.8)		5 (27.8)	5 (25.0)
History of diabetes, # (%)		17 (34.7)	25 (51.0)		10 (50.0)	8 (40.0)
Smoking habits, # (%)						
Non-smokers		29 (61.7)	23 (51.1)		12 (63.2)	13 (65.0)
Current smokers		13 (27.7)	16 (35.6)		6 (31.6)	5 (25.0)
Ex-smokers		5 (10.6)	6 (13.3)		1 (5.3)	2 (10.0)
Alcohol consumption, # (%)						
Non-drinkers		22(46.8)	20(44.4)		11(57.9)	9(45.0)
Current drinkers		23(48.9)	19(42.2)		7(36.8)	8(40.0)
Ex-drinkers		2(4.3)	6(13.3)		1(5.3)	3(15.0)

*BMI* body mass index, *FPG* fasting plasma glucose, *HbA1c* glycated hemoglobin, *HDL* high-density lipoprotein, *LDL* low-density lipoprotein, *TG* triglyceride, *TC* total cholesterol, *Systolic BP* systolic blood pressure, *Diastolic BP* diastolic blood pressure, *Control* healthy control group, *DM* T2DM without DR participants, *DR* T2DM patients with DR.

Continuous data obeying normal or similar normal distribution were described as mean ± standard deviation (SD) and variance analysis of randomized block design or the paired t-test was applied to compare the differences between the three/two groups. Otherwise, median (1st quartile, 3rd quartile) and Friedman M or Wilcoxon signed-rank tests were used. Categorical data were presented as a number of cases (%) and the McNemar-Bowker test was utilized to compare the differences between the groups.

### Data preprocessing and feature screening

The flowchart of data preprocessing and feature screening from the metabolomics data could be found in Fig. 1A. Among a total of 532 metabolites detected by UPLC-MS/MS system, 483 features had CV under 30% in the QC samples, and 449 with missing values less than 20%. In the end, 380 features were included in the final data analyzes after the screening via the variance filtering and mutual information methods.

### Construction and evaluation of machine learning model

Table S1 shows the classification results of the parameter optimization model based on the Sklearn package in Python. It can be seen that RF and XGBoost, which are typical algorithms under the framework of Bagging and Boosting in the integrated algorithm, had excellent classification capabilities. Accuracy, precision, recall, and F1-score were all above 95%. NNs also had excellent classification performance. As typical representatives of interpretable machine learning, the model performance of DT and LR was close to RF, XGBoost, and NNs, with each evaluation index higher than 90%. KNN, GNB, and SVM were slightly inferior.

The accuracy of the 10-fold cross-validation of eight machine learning models was tested by Friedman. As shown in Fig. 1B, the accuracy of the models was not the same (*P* < 0.05). The Nemenyi test was further used for the pair-wise comparison of the accuracy of the eight models. From the heat map of the model comparison, it could be seen that the model performance of KNN and GNB was inferior to RF, XGBoost, and NNs (*P* < 0.05). There was no significant difference among the other models (*P* > 0.05).

Considering the interpretability of the model, DT and LR have inherent advantages, and in this study, the performance of these two models was not inferior to other models. Compared with LR, DT model was more concise. Therefore, DT was selected for further analysis considering the performance, interpretability, and simplicity of the model.

### Constructing a decision tree and verification of prediction accuracy

As we all know, the parameter adjustment strategy has a huge influence on the DT, and the correct strategy is the core of optimizing the decision tree algorithm. First of all, we used the hyper-parameter learning curve to determine the maximum depth of the tree. As shown in Fig. 1(C), when the parameter max_depth = 2, the model had the highest accuracy. Furtherly, we used grid search technology to determine the optimal parameters of the tree model (criterion = ‘gini’, min_samples_leaf = 1, min_samples_split = 2).

It could be seen from Fig. 1(D) that the root node of DT was 2-pyrrolidinone. Participants with 2-pyrrolidinone peak areas over 9,910,000.0 were divided into the healthy control group. The second node for the branch of 2-pyrrolidinone under 9,910,000.0 was thiamine triphosphate (ThTP). All participants with ThTP peak area under 24350.0 were classified as DR patients, otherwise, they were classified as DM patients.

Application of the CART DT yielded good discrimination of DR in the training set (accuracy, 94.6%). To evaluate the generalization ability of this DT, we used the hold-out and 10-fold cross-validation to assess it at the same time (Table S2). We found that the accuracy of DT evaluated by the hold-out and cross-validation were 93.3% and 94.3%, respectively. Precision, recall, and f1-score were also higher than 90%.

### Combination of clinical and metabolic biomarkers for DR recognition

Identifying DR cases from T2DM patients efficiently was the major objective of this study. As shown in Fig. 1D, ThTP could achieve this goal well, and the correlation analysis once again verified this result (Table S3). After adjusting for SBP and the duration of diabetes, the association between ThTP and DR was significant. With each increase in standard deviation (SD), the probability of DR occurrence was reduced by 100% [OR:0.00, 95%CI (0.00, 0.03); *P* < 0.001]. According to the cutoff points found by the DT model, the probability of developing DR in people with ThTP level less than 24350 was 311.32 times that in those whose serum ThTP were above or equal to 24350 [OR: 311.32, 95%CI (32.75, 2959.78); *P* < 0.001]. In the multivariable analysis, the probability of occurrence of DR increased by 23% for every year extension of the disease duration [OR: 1.23, 95%CI (1.03, 1.48); *P* = 0.023]; and for every additional SD, increased by 228% [OR: 3.28, 95% CI (1.05, 10.27); *P* = 0.042]. According to the cutoff point found by the cubic spline curve (Fig. S2), the probability of DR in people with a disease course longer than 10 years is 22.95 times that of a population shorter than 10 years [OR: 22.95, 95% CI (1.73,304.65); *P* = 0.018].

**Fig. 2:**
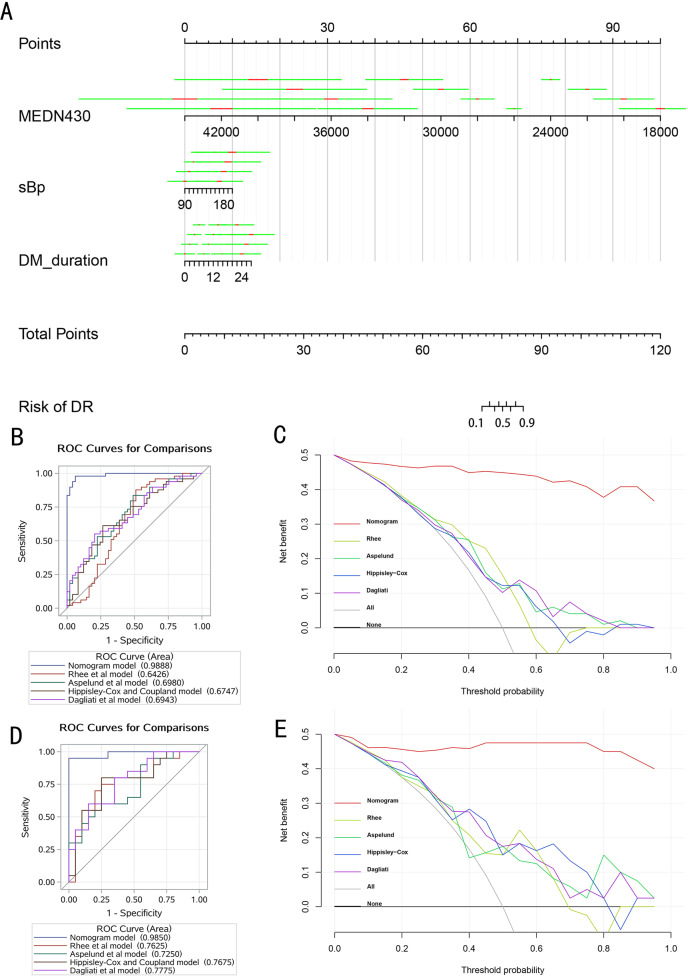
Development and validation of the nomogram model. Developed nomogram for diabetic retinopathy (**A**), and the ROC curve and decision curves analysis curve of the Nomogram model, Rhee et al. model, Aspelund et al. model, Hippisley-Cox and Coupland model, and Dagliati et al. model in the training set (**B**, **C**) and testing set (**D**, **E**). Notes: nomogram model, thiamine triphosphate, systolic blood pressure, duration of diabetes; Rhee et al. model, glutamine/glutamate ratio; Aspelund et al. model, gender, systolic blood pressure, duration of diabetes and glycated hemoglobin; Hippisley-Cox and Coupland model, age, BMI, systolic blood pressure, cholesterol/high-density lipoprotein ratio, glycated hemoglobin; Dagliati et al. model, age, gender, duration of diabetes, BMI, glycated hemoglobin, hypertension, smoke; none, net benefit when all patients are considered as not having the outcome (diabetic retinopathy); all, net benefits when all patients are considered as having the outcome. The preferred model is the model with the highest net benefit at any given threshold. Abbreviations: MEDN430 thiamine triphosphate, sBp systolic blood pressure, DM_duration duration of diabetes.

Although the SBP did not reach statistical significance in the multivariable analysis, considering its clinical importance, we still included it in the model analysis. Then, we combined the above 3 biomarkers to develop a screening model and displayed it as a nomogram diagram (Fig. 2A) in the training set. The calibration curve of the nomogram to predict the DR risk in T2DM patients showed nice agreement with a non-significant Hosmer–Lemeshow Chi-square of 2.68 (*P* = 0.953) and 3.99 (*P* = 0.858) in the training and testing set (Fig. S3), respectively. These results all show that the model had good consistency.

Several models for early identification or diagnosis of DR have been reported [20–23]. The ability of each model was assessed by AUC (Table 2, Fig. 2). Among them, the AUCs for the nomogram in either the training set (AUC, 0.989; 95% CI, 0.974–1.000) or the testing set (AUC, 0.985; 95% CI, 0.954–1.000) were all significantly higher than those of previous models (*P* < 0.05). In addition, the cutoff value of the total points of the model in the training set was 79.11. According to the cutoff value, DM patients could be divided into high-risk and low-risk groups. And the sensitivity was 97.96%, the specificity was 93.88%, the accuracy was 95.92%, the positive predictive value was 94.12%, the negative predictive value was 97.87%, and the Youden index was 0.92 (Table 2). The model still had excellent classification ability in the testing set. The sensitivity, specificity, accuracy, positive predictive value, negative predictive value, and Youden index were 95.00%, 100.00%, 97.50%, 100.00%, 95.24%, and 0.95, respectively (Table 2). As shown in Fig. 2, whether in the training set or testing set, the nomogram model performed outstandingly in various predictors regardless of the threshold, which ensured maximum clinical benefit.

**Table 2:** Comparison of the predictive ability of the Nomogram model and models constructed in previous studies.

Model	AUC	AUC (95%CI)	Sensitivity (%)	Specificity (%)	Precision (%)	Positive predictive value (%)	Negative predictive value (%)	Youden’s index
Training set								
Nomogram model	0.99	0.97, 1.00	97.96	93.88	95.92	94.12	97.87	0.92
Rhee et al. model	0.64	0.53, 0.76	87.76	48.98	68.37	63.24	80.00	0.37
Aspelund et al. model	0.70	0.59, 0.80	83.67	51.02	67.35	63.08	75.76	0.35
Hippisley-Cox and Coupland model	0.67	0.57, 0.78	61.22	73.47	67.35	69.77	65.45	0.35
Dagliati et al. model	0.69	0.59, 0.80	55.10	79.59	67.35	72.97	63.93	0.35
Testing set								
Nomogram model	0.99	0.96, 1.00	95.00	100.00	97.50	100.00	95.24	0.95
Rhee et al. model	0.76	0.61, 0.92	75.00	75.00	75.00	75.00	75.00	0.50
Aspelund et al. model	0.73	0.57, 0.88	55.00	80.00	67.50	73.33	64.00	0.35
Hippisley-Cox and Coupland model	0.77	0.62, 0.92	80.00	75.00	77.50	76.19	78.95	0.55
Dagliati et al. model	0.78	0.63, 0.92	80.00	65.00	72.50	69.57	76.47	0.45

Nomogram model contains thiamine triphosphate, systolic blood pressure, and duration of diabetes; Rhee et al. model contains glutamine/glutamic acid ratio; Aspelund et al. model contains sex, systolic blood pressure, duration of diabetes, and glycated hemoglobin; Hippisley-Cox and Coupland model contains sex, BMI, systolic blood pressure, cholesterol/high-density lipoprotein ratio, and glycated hemoglobin; Dagliati et al. model contains age, sex, duration of diabetes, BMI, glycated hemoglobin, hypertension, and smoking.

In particular, we selected DR patients with different degrees from the DR group for sensitivity analysis. The above-established nomogram model still had an excellent ability in distinguishing diabetic without DR participants and patients with mild DR, and AUCs were 0.997 (95% CI, 0.987–1.000) and 1.000 (95% CI, 1.000–1.000) in the training set and testing set (Fig. S4). Similarly, the AUCs for moderate DR were 1.000 (95% CI, 1.000–1.000) and 0.964 (95%CI, 0.889–1.000), and the severe is 0.968 (95%CI, 0.925–1.000) and 1.000 (95% CI, 1.000–1.000).

## Discussion

From the serum metabolomic analysis of non-diabetic controls and diabetic patients with and without DR, a lot of metabolites were selected as candidate biomarkers through standardized data preprocessing, and a DT model (including 2-pyrrolidinone and ThTP) was constructed based on the CART algorithm (Fig. 1D). These two metabolites belong to the glutamate metabolism pathway, and glutamate metabolism is known to be closely related to insulin resistance and secretion [20, 24]. Furthermore, by integrating clinical and metabolomics indicators, including diabetes duration, SBP, and ThTP, the constructed nomogram model could well select DR patients from T2DM patients and had a proper calibration.

### Comparison with other studies on DR

The occurrence and development of disease is a nonlinear dynamic process through health status, pre-disease, and disease stage [11, 25]. Although no typical clinical symptoms and signs occurred in the early stage of the disease, significant changes still occurred in the pathophysiology [26]. There is a period of time between the development of diabetes and the first clinical signs of DR. Therefore, early detection of subclinical DR can provide timely identification and treatment for patients at higher risk of DR progression. Several models to early detect the risk of DR have been developed, such as the model of Rhee et al. [20], Aspelund et al. [23], Hippisley-Cox and Coupland [22], and Dagliati et al. [21]. In particular, Rhee and his colleagues used the ratio of glutamine and glutamic acid as a predictor of DR risk. Similarly, our model mainly used substances related to glutamate metabolism for early identification, but our model had a higher degree of discrimination and calibration (Fig. 2). A common feature of the other three models is that they only contain demography and clinical characteristics. This might be the main reason for their unsatisfactory prediction results.

### Biological plausibility and implications

We could see from our DT model that 2-pyrrolidone was a perfect biomarker to distinguish diabetic patients from healthy people, and at the same time, ThTP could screen DR patients from diabetic patients.

2-pyrrolidone is the lactam cyclization product of γ-aminobutyric acid (GABA) [27], which can be produced in the human body through various routes. It is recognized that the main route of GABA formation is through the decarboxylation of glutamic acid, which then spontaneously generates 2-pyrrolidone [28]. Studies have shown that 2-pyrrolidone had free radical scavenging activity [29]. ThTP, a triphosphorylated derivative of vitamin B_1_, is a non-coenzyme form of thiamine [30]. However, the widespread existence of ThTP from prokaryotes to mammals suggests that it may play a fundamental role in cell metabolism or cell signal transduction [31]. Besides, ThTP acted as a glutamate dehydrogenase (GLDH) activator at micromolar concentrations [32] and was considered to be an allosteric activator of GLDH [33], and this effect has been supported in cell experiments [34]. In addition, some people thought that ThTP had a specific neurophysiological effect [35], and more and more evidence showed that retinal neurodegeneration was an early event in the pathogenesis of DR and might be involved in the development of microvascular abnormalities [36].

As shown in Fig. S5, 2-pyrrolidone and ThTP were related to glutamate metabolism. Glutamate metabolism is connected to various cell functions, such as protein synthesis, pancreatic β-cell insulin secretion, liver, and kidney gluconeogenesis, and neurotransmitter synthesis [37]. In particular, glutamate concentration is also one of the most critical indicators of the diabetic retina [20]. Some studies have shown that the accumulation of glutamate in diabetes can lead to the development of DR [38–40]. Furthermore, from the healthy control, DM to DR groups, ThTP and the substances in the tricarboxylic acid cycle (succinic acid, fumaric acid, and citramalic acid) all showed a “∪“ or “∩“ shape. We speculate that at high glucose levels, a decrease in ThTP predicted changes in early symptoms of the retina. Although the direct relationship between 2-pyrrolidone and ThTP and DR has not been well studied, we could be cautious that the level of these two substances may be closely related to the occurrence and development of DR, and used as a valid biomarker for early screening of DR.

### Strengths and limitations

Our results have some notable differences from previous studies. With the unprecedented accumulation of information, the relevance of machine learning is increasing, and new algorithms and tools are constantly emerging. According to the no free lunch theorem [41], a universal optimal performance optimization algorithm is rare. So, we developed a predictive model mainly based on the data attributes and experimental objectives in the present study. Furthermore, as many machine learning model was unexplainable, we applied CART DT, an interpretable model, for feature selection and model construction, which not only gave full play to the advantages of machine learning in dealing with high-dimensional data but also made our model easy to be understood and accepted. It also decreased the problems of traditional statistical methods for data distribution with the analysis of metabolomics data and avoided the inflation of the type I error with the multiple comparisons. At the same time, standardized data preprocessing procedures and parameter adjustment methods could solve the major shortcoming of the overfitting in the CART DT model. In practice, the performance of the decision tree model in our study had been improved. In addition, a PSM approach was also applied to adjust for influences induced by some demographic and clinical features as much as possible. In statistics, PSM was believed to be able to highly decrease the impacts due to some potential confounding factors in the findings. Finally, the performance of the constructed model was comprehensively assessed by sufficient evaluations of the discrimination, calibration, and clinical values. We believed that the proposed nomogram model (including diabetes duration, systolic blood pressure, and ThTP) could distinguish DR cases from T2DM patients precisely and efficiently, and was convenient for clinical application.

Limitations also existed in this study. As could be found in the manuscript, few PDR patients were also included in the DR cases, which might partly increase the uncertainty of several biomarkers unsuitable for DR early identification to some extent. However, in the sensitivity analysis only including NPDR and T2DM patients without DR, the nomogram model we proposed still showed outstanding capabilities in both the training and testing sets. In addition, our results were based on the cross-sectional analysis, which made it difficult to explain the potential causal relationships. Nevertheless, it should be noted that the objective of this study was to propose an ideal metabolomics-based predictive model for detecting DR cases among T2DM patients effectively rather than proving the causal links between the initiation of DR and metabolites. It would be brilliant if further studies could not only confirm our findings but also clarify the metabolic mechanism of DR initiation and development.

## Conclusion

In summary, with an extensively targeted serum metabolomics analysis and interpretable machine learning model, this study suggests that 2-pyrrolidone and ThTP are the major characteristics of serum metabolites of T2DM and can not only precisely distinguish T2DM patients from non-diabetic participants but also effectively detect DR cases from T2DM patients without DR. ThTP can be served as an acceptable specific sensitive biomarker of DR occurrence. Furthermore, a machine learning-derived nomogram model, integrated with systolic blood pressure, duration of diabetes, and ThTP, has an excellent ability to distinguish DR from T2DM patients precisely and efficiently. Our findings provide new insight into the clinical and public health policy relevance of precise administration and control of DR.

## Supplementary information

Supplemental material

## Data Availability

The datasets used and/or analyzed during the current study are available from the corresponding author on reasonable request.
