# Supplementary material for: Interpretable machine learning-derived nomogram model for early detection of diabetic retinopathy in type 2 diabetes mellitus: a widely targeted metabolomics study
Source: Nutr Diabetes. 2022 Aug 5;12:36. doi: 10.1038/s41387-022-00216-0 (PMC9355962; doi:10.1038/s41387-022-00216-0)
Supplement: Supplementary file 1 — Supplemental material [file 41387_2022_216_MOESM1_ESM.docx]

**Table S1.** Performance evaluation of machine learning models after parameter optimization

|  | Accuracy | Precision | | | Recall | | | F1-score | | |
| --- | --- | --- | --- | --- | --- | --- | --- | --- | --- | --- |
|  |  | macro^ξ^ | micro^ζ^ | weighted^#^ | macro^ξ^ | micro^ζ^ | weighted^#^ | macro^ξ^ | micro^ζ^ | weighted^#^ |
| KNN | 0.868 | 0.876 | 0.868 | 0.876 | 0.868 | 0.868 | 0.868 | 0.865 | 0.868 | 0.865 |
| GNB | 0.898 | 0.921 | 0.898 | 0.921 | 0.898 | 0.898 | 0.898 | 0.898 | 0.898 | 0.895 |
| LR | 0.975 | 0.979 | 0.975 | 0.979 | 0.975 | 0.975 | 0.975 | 0.974 | 0.975 | 0.974 |
| DT | 0.943 | 0.961 | 0.943 | 0.961 | 0.943 | 0.943 | 0.943 | 0.939 | 0.943 | 0.939 |
| RF | 0.995 | 0.996 | 0.995 | 0.996 | 0.995 | 0.995 | 0.995 | 0.995 | 0.995 | 0.995 |
| XGBoost | 0.967 | 0.974 | 0.967 | 0.974 | 0.967 | 0.967 | 0.967 | 0.966 | 0.967 | 0.966 |
| NNs | 0.986 | 0.988 | 0.986 | 0.988 | 0.986 | 0.995 | 0.986 | 0.986 | 0.986 | 0.986 |
| SVM | 0.903 | 0.926 | 0.903 | 0.926 | 0.903 | 0.903 | 0.903 | 0.900 | 0.903 | 0.900 |

*^ξ^macro-average performance, means the way in which we averagingly computed the unweighted measure per-class;*

*^ζ^micro-average performance, represents the way in which the corresponding elements of each confusion matrix were averaged, and the measure was then calculated;*

*^#^weighted performance, represents the approach in which the corresponding elements of each confusion matrix were separately calculated first, and obtained the averaged measure weighted by the weight of each category.*

*Model parameter settings:*

*KNN: n_neighbors=3, weights="distance", p=1;*

*GNB: standardization;*

*LR: First of all, the embedding method was used for feature selection (105 features are retained). penalty="l1", solver="liblinear", C=0.156, max_iter=24, random_state=420;*

*DT: max_depth=2, criterion="gini", random_state=12345, min_samples_leaf=1, min_samples_split= 2;*

*RF: n_estimators=166, random_state=123, max_depth=8, min_samples_leaf=1, min_samples_split=2, max_features=19;*

*XGBoost: n_estimators=11, learning_rate=0.3, subsample=1, random_state=123, booster="gbtree", max_depth=4**;*

*NNs: standardization, hidden_layer_sizes=(100,30), activation="relu", max_iter = 2000, random_state=420;*

*SVM: random_state=123, kernel="linear", C=0.01.*

**Table S2.** Performance evaluation of decision tree (DT) model

| Sets | Accuracy | Precision | | | Recall | | | F1-score | | |
| --- | --- | --- | --- | --- | --- | --- | --- | --- | --- | --- |
|  |  | macro^ξ^ | micro^ζ^ | weighted^#^ | macro^ξ^ | micro^ζ^ | weighted^#^ | macro^ξ^ | micro^ζ^ | weighted^#^ |
| Hold-out |  |  |  |  |  |  |  |  |  |  |
| Train set | 0.946 | 0.946 | 0.950 | 0.950 | 0.946 | 0.946 | 0.946 | 0.946 | 0.945 | 0.945 |
| Test set | 0.933 | 0.933 | 0.944 | 0.944 | 0.933 | 0.933 | 0.933 | 0.933 | 0.933 | 0.933 |
| Cross validation | 0.943 | 0.961 | 0.943 | 0.961 | 0.943 | 0.943 | 0.943 | 0.939 | 0.943 | 0.939 |

*^ξ^macro-average performance, means the way in which we averagingly computed the unweighted measure per-class;*

*^ζ^micro-average performance, represents the way in which the corresponding elements of each confusion matrix were averaged, and the measure was then calculated;*

*^#^weighted performance, represents the approach in which the corresponding elements of each confusion matrix were separately calculated first, and obtained the averaged measure weighted by the weight of each category.*

**Table S3.** Screening out risk factors for DR by conditional logistic regression in the training set

| Risk factors | n | DR,#(%) | Univariate | | Multivariable | | |
| --- | --- | --- | --- | --- | --- | --- | --- |
|  |  |  | OR (95%CI) | P | OR (95%CI) | P | |
| Thiamine triphosphate | | | | | | |  |
| Per 1 unit |  |  | 1.00(1.00,1.00) | <0.001 | 1.00(1.00,1.00) | 0.001 | |
| Per SD |  |  | 0.01(0.00,0.04) | <0.001 | 0.00(0.00,0.03) | <0.001 | |
| Cut-off value |  |  |  |  |  |  | |
| ≥24350 | 55 | 7(12.70) | 1.00(1.00,1.00) | Ref. | 1.00(1.00,1.00) | Ref. | |
| <24350 | 43 | 42(97.70) | 288.00(34.03,2437.63) | <0.001 | 311.32(32.75,2959.78) | <0.001 | |
| Systolic BP | | | | | | |  |
| Per 1 mmHg |  |  | 1.03(1.00,1.05) | 0.031 | 1.04(0.96,1.13) | 0.349 | |
| Per SD |  |  | 1.50(0.96,2.34) | 0.077 | 1.87(0.46,7.57) | 0.382 | |
| Clinical criteria | |  |  |  |  |  | |
| <140 | 70 | 33(47.10) | 1.00(1.00,1.00) | Ref. | 1.00(1.00,1.00) | Ref. | |
| ≥140 | 28 | 16(57.10) | 1.50(0.62,3.62) | 0.372 | 8.08(0.24,277.70) | 0.247 | |
| Cut-off value |  |  |  |  |  |  | |
| <130 | 51 | 18(35.30) | 1.00(1.00,1.00) | Ref. | 1.00(1.00,1.00) | Ref. | |
| ≥130 | 47 | 31(66.00) | 3.55(1.54,8.17) | 0.003 | 2.62(0.30,22.75) | 0.382 | |
| Duration of diabetes | | | | | | |  |
| Per 1 year |  |  | 1.10(1.03,1.18) | 0.007 | 1.23(1.03,1.48) | 0.023 | |
| Per SD |  |  | 1.77(1.14,2.75) | 0.011 | 3.28(1.05,10.27) | 0.042 | |
| Cut-off value |  |  |  |  |  |  | |
| <10 | 45 | 16(35.60) | 1.00(1.00,1.00) | Ref. | 1.00(1.00,1.00) | Ref. | |
| ≥10 | 53 | 33(62.30) | 2.99(1.31,6.83) | 0.009 | 22.95(1.73,304.65) | 0.018 | |

*SD: standard deviation.*

*Cut-off value was determined using cubic spline curve or decision tree.*


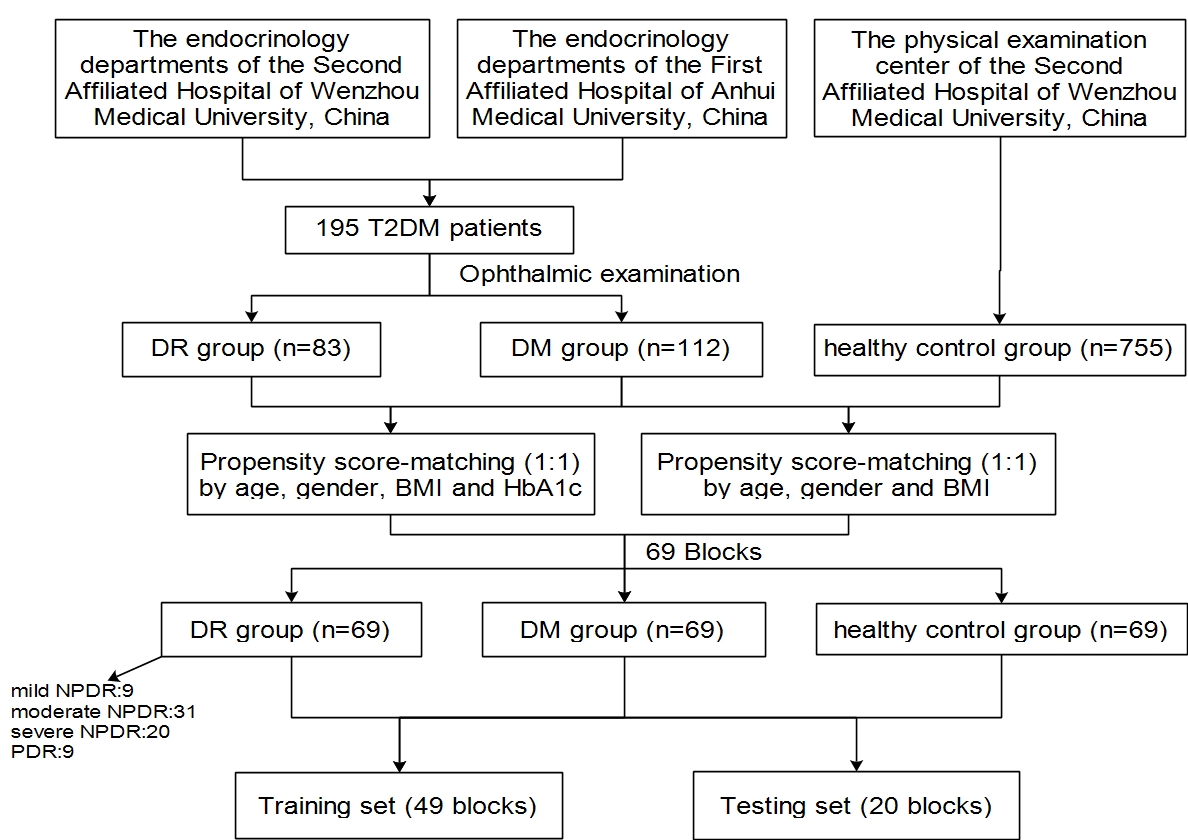


**Fig S1.** Study flowchart of the propensity score-matched participants

*Abbreviations: T2DM, Type 2 diabetes; DR, diabetic retinopathy; DM, diabetes mellitus without DR; NPDR, non-proliferative diabetic retinopathy; PDR, proliferative diabetic retinopathy.*

*
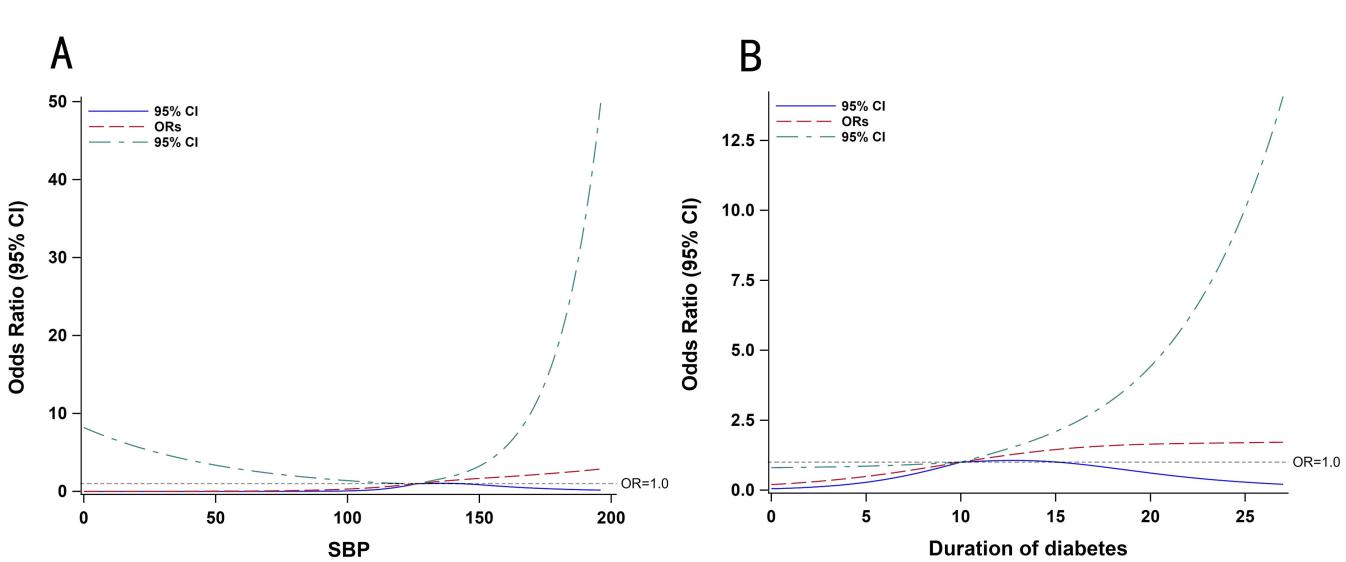
*

**Fig S2.** The relation of SBP (A) and duration (B) with the risk of DR

*
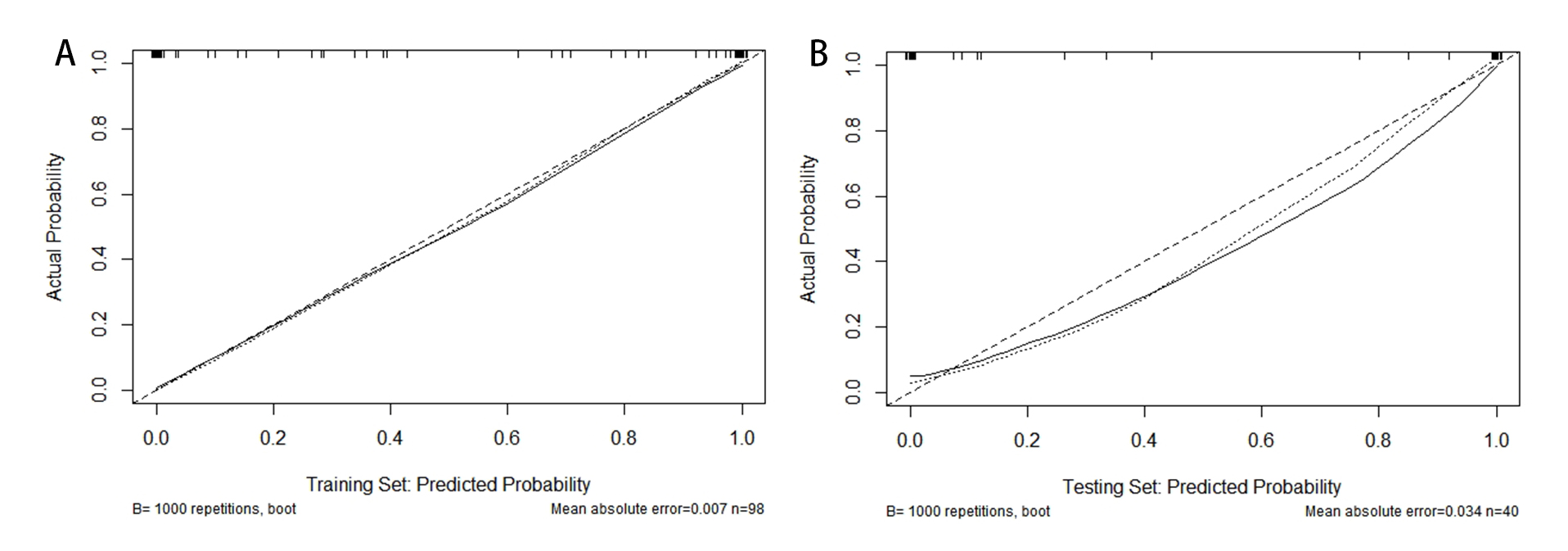
*

**Fig S3.** Calibration curves of the DR incidence risk nomogram prediction in the array

*Notes: The x-axis represents the predicted incidence risk. The y-axis represents the actual diagnosed DR. The diagonal dotted line represents a perfect prediction by an ideal model. The solid line represents the performance of the nomogram; a closer fit to the diagonal dotted line represents a better prediction.*

*
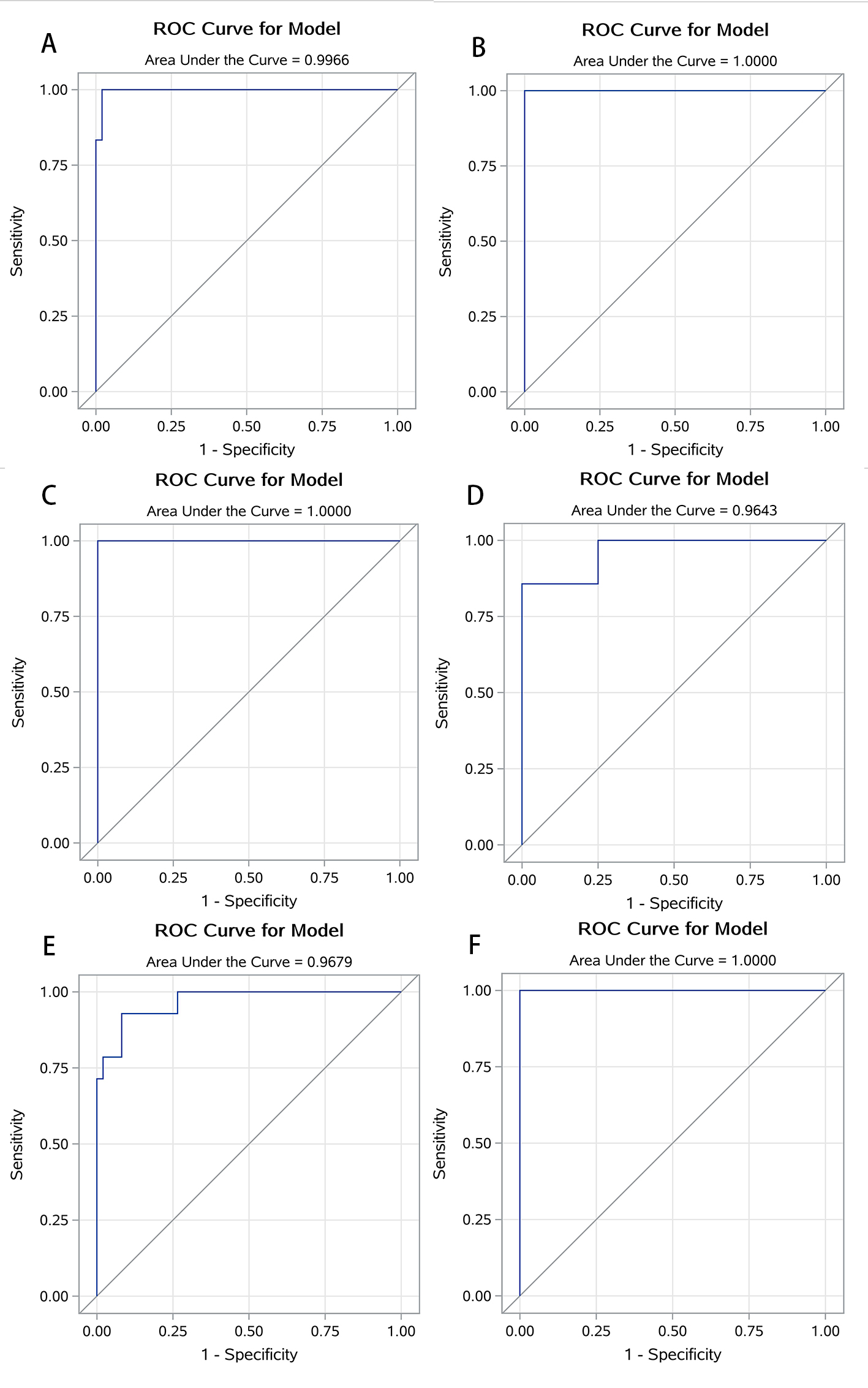
*

**Fig S4.** Sensitivity analysis of patients with DM and mild/moderate/severe DR: the ROC curve of the nomogram model in the training set (A, C, E) and testing set (B, D, F)

*Notes: mile, A and B; moderate, C and D; severe, E and F.*

*
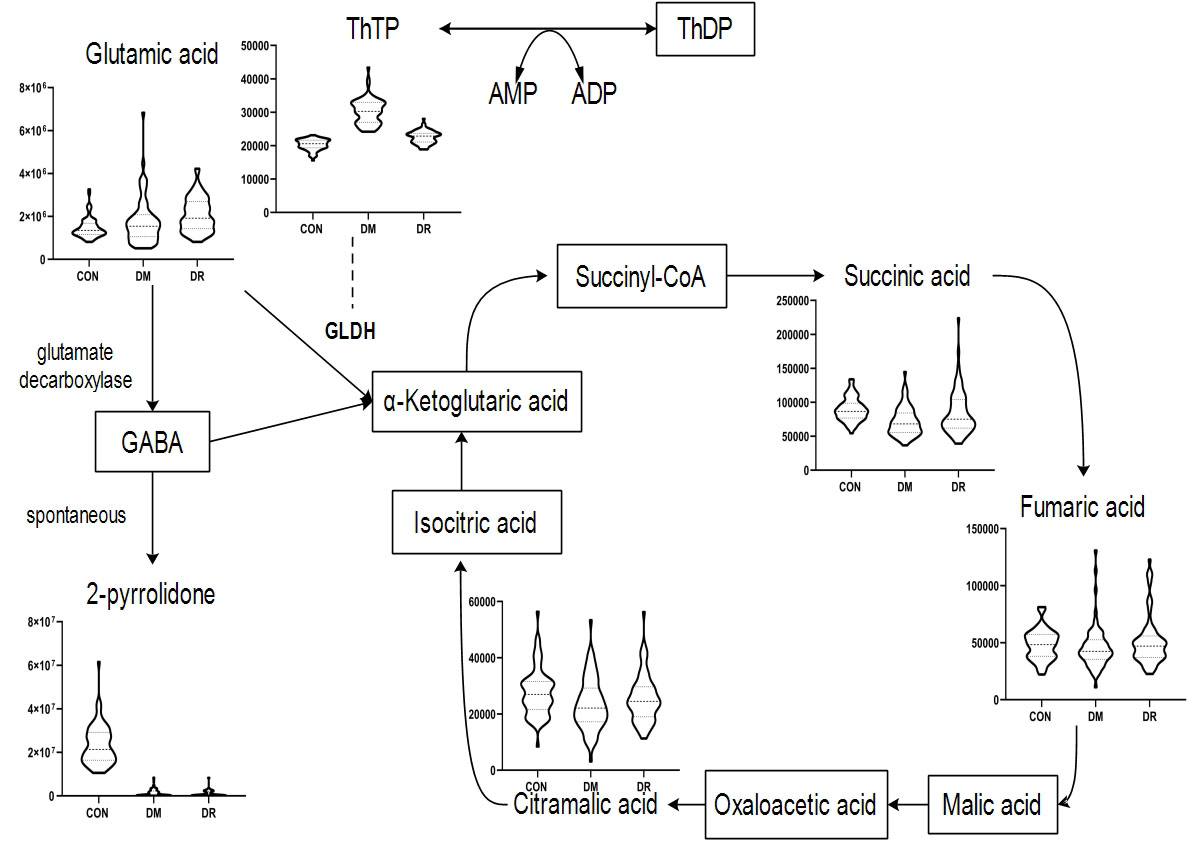
*

**Fig S5.** A schematic diagram of a proposed metabolic pathway

*Abbreviations: ThTP, thiamine triphosphate; ThDP, thiamine diphosphate; AMP, adenosine monophosphate, ADP, adenosine diphosphate; CON, healthy control group; DR, diabetic retinopathy group; DM, diabetes mellitus without DR group; GABA, γ-aminobutyric acid; GLDH, glutamate dehydrogenase.*
